# Food waste in Indian households: status and potential solutions

**DOI:** 10.1007/s11356-023-31034-1

**Published:** 2023-11-15

**Authors:** Samant Shant Priya, Sushil Kumar Dixit, Sajal Kabiraj, Meenu Shant Priya

**Affiliations:** 1 Lal Bahadur Shastri Institute of Management (LBSIM), New Delhi, Delhi, India; 2 Lal Bahadur Shastri Institute of Management, New Delhi, India; 3 School of Business, Design and Technology, Häme University of Applied Sciences Ltd. (HAMK), Valkeakoski, Finland; 4 School of Business, Galgotias University, Greater Noida, India

**Keywords:** Eradicating hunger, Responsible consumption and production, SDG 2, SDG 12, Qualitative research, Phenomenology

## Abstract

According to a report by the Food and Agriculture Organization (FAO), India had the highest number of undernourished people in the world in 2020. The COVID-19 pandemic further exacerbated the problem of world hunger (WHO 2021). According to the Food Waste Index Report, 2021, by United Nations Environment Programme (UNEP) and The Waste and Resources Action Programme (WRAP), 931 million tonnes of food waste was produced in 2019, with households accounting for 61%, food services for 26%, and retail for 13%. The report estimates that Indian households generate 50 kg of food waste per capita per year, resulting in total of 68,760,163 tonnes annually. This study aimed to investigate the reasons for food waste in Indian households and potential solutions to minimize or control food waste using interpretive phenomenological analysis. Reasons for food waste include miscalculations in meal preparation, a lack of appreciation for food, and a weakening of traditional Indian value systems. The study identified two potential solutions for controlling or eliminating food waste: exploring alternative methods of food consumption and enhancing the family culture surrounding food. The study results could potentially guide policymakers and planners in designing policies to address the problem of food waste in Indian households.

## Introduction

The United Nations (UN) introduced the Sustainable Development Goals (SDGs) in 2015 to address poverty and environmental concerns, and promote peace and prosperity (UN 2015). SDG 12 specifically focuses on responsible consumption and production, with goal 12.3 aiming to reduce global per capita food waste by half by 2030. The Zero Hunger Challenge, launched by the UN Secretary-General Ban Ki-moon in 2012, aims to eliminate food insecurity and malnutrition while building sustainable food systems (UN 2012). If current trends continue, however, the number of people affected by hunger is expected to surpass 840 million by 2030. Minimizing food losses during production, storage, transport, and consumption, empowering consumer choice, and ensuring commitment by producers, retailers, and consumers are all crucial to meeting the Zero Hunger Challenge (James et al. 2020). Chronic malnutrition has increased in many parts of the world including most Indian states, and malnourished children are more vulnerable to illness and disease (Bhargava and Bhargava 2021; FAO 2021; World Bank 2020). The Executive Director of the UNEP, Inger Andersen, emphasized the significance of reducing food waste in addressing global issues such as climate change, biodiversity loss, and pollution and waste, and that it is essential for businesses, governments, and individuals to play their part in minimizing food waste to combat these challenges (UNEP 2020).

The terms food loss, food waste, biowaste, and kitchen waste are used interchangeably (Gjerris and Gaiani 2013). Thyberg and Tonjes (2016) classify food waste into two types: food loss and food waste. Food loss refers to any edible food that goes uneaten at any stage, including crops left in the field, food that spoils during transit, and food that does not make it to a store, in addition to uneaten food in households and businesses. Food waste, on the other hand, refers to food that is discarded or uneaten after it has been purchased or served, and it can occur at any stage of the food chain. Examples of food waste include unconsumed meals in restaurants, leftovers from home-cooked meals, and spoiled food (FoodPrint 2018). The European Commission (2014) categorizes food waste into three types: food losses, unavoidable food waste, and avoidable food waste.

The UN Food and Agriculture Organization (FAO) defines food waste and food loss as:

*“a decrease in quantity or quality of food. Food waste is part of food loss and refers to discarding or alternative (non-food) use of food that is safe and nutritious for human consumption along the entire food supply chain, from primary production to end household consumer level”* (FAO 2011).

According to the Global Hunger Index (2021), India ranks 101 out of 116 countries measured in 2021. The amount of food waste in India is equivalent to the amount of food consumed by the United Kingdom (Sinha and Tripathi 2021). In light of these findings, this study aims to qualitatively investigate the factors that contribute to food waste in Indian households and propose potential solutions to mitigate the issue. While extensive literature on food waste exists, most of it focuses on the Western world, leaving a knowledge gap regarding developing economies such as India. Food waste is a complex phenomenon that is heavily influenced by contextual factors like value system, affordability, availability, value system etc. Additionally, it is frequently attributed to individual cognitive and behavioural choices. However, limited research has been conducted to examine the lived experiences of families in terms of food and its waste. Therefore, this study seeks to explore the core aspects and lived experiences of food waste in Indian households.

The research questions that guide this exploration include: "What is the experience of food waste among Indian households?"; "What are the factors that contribute to food waste in Indian households?"; and "What is the best solution for reducing food waste in Indian households?" By addressing these questions, the study hopes to provide valuable insights into how to minimize or control food waste in Indian households, which can inform policymakers in designing effective policies and programs.

## Method

The present study adopts a qualitative research approach to investigate the factors affecting food waste in Indian households. Qualitative research aims to reveal the meaning and experience of people's lives and social environments (Fossey et al. 2002), making it a suitable method for exploring complex issues such as food waste. Qualitative researchers use naturalistic inquiry to inductively examine real-world environments and develop rich narrative descriptions (Patton 2005). In this study, we employ phenomenology as the specific qualitative research method, which focuses on the lived experiences of a few individuals and their perceptions of these experiences to generate significant insights (Thompson 1997). Creswell and Poth (2016) suggest that phenomenology can be used to investigate complex topics with limited literature. Through semi-structured interviews, we aim to explore the phenomenon of food waste experienced in Indian households within the context of their food consumption. Our use of qualitative analysis facilitates a thorough investigation of the problem and may lead to the development of a theoretical framework (Miles and Huberman 1994). To understand the factors that contribute to food waste, we analysed participants' perspectives gathered through semi-structured interviews.

For the current study, respondents from the National Capital Region (NCR) in India were selected. This region was selected due to its diverse population in terms of income, age, education, religious and cultural background, as well as regional affiliations. The researcher collected data from respondents from Delhi, Noida, Gurgaon, and Ghaziabad with varied gender, age, and occupational distribution. All interviews were recorded except for five participants who declined to be recorded, and the researcher simultaneously made notes during the conversations. Initially, twenty samples were recruited for the study, but the researchers ceased data collection at the thirteenth interview, as the emergence of novel data and themes had ceased. This was considered indicative of reaching saturation point, which is regarded as the ‘gold standard’ in qualitative inquiry (Fusch and Ness 2015; Guest et al. 2006). In this study, data saturation was reached at thirteen interviews, which is consistent with previous studies by Guest et al. (2006) and Francis et al. (2010), where data saturation was reached at twelve and seventeen interviews, respectively.

To investigate the lived experiences of food waste in Indian households, semi-structured, open-ended, and in-depth interviews were conducted. Prior to data collection, an initial conceptual framework, interview guide, and a set of research questions were developed following Miles and Huberman's (1994) recommendations. The study’s objectives and adaptive decision-making model conceptual framework were used to define the scope of the investigation and prevent superfluous data accumulation. The interview guide consisted of twelve questions that focused on food waste, feelings about food waste, and the reasons for and possible solutions to food waste. Respondents were approached in their homes and given a general overview of the study's purpose before commencing the interview. The interviews, which lasted between 20 and 65 minutes, were conducted in the local language and recorded for later transcription and translation into English. The transcripts were reviewed for accuracy, ensuring that the participants' viewpoints were not lost in translation.

To protect the privacy of participants, they were informed that their interviews and collected information would be kept confidential. The researchers obtained consent from the participants for using the collected data in academic publications. To ensure the participants' anonymity, the researchers used pseudonyms and removed any data that could reveal their identities The data was stored in a password-protected folder, and participants were instructed to contact the researchers if they felt uncomfortable during or after the interviews.

## Results

The researchers employed interpretative phenomenological analysis (IPA) as recommended by Smith et al. (2022) to analyze the interview transcripts. The analysis began with a comprehensive review and re-reading of the transcripts to gain a general understanding of the participants' stories and to identify any emerging themes or novel information. Titles were assigned to the emerging themes, and the themes were abstracted while preserving the participants' original accounts. The subsequent step involved documenting the emerging themes and consolidating them to obtain the core of the participants' experiences with food waste. The authors then independently identified clusters of related themes across the transcripts, which were compiled into a list of master themes to ensure face validity. Finally, all authors revised the master and subordinate themes to ensure that the analysis accurately reflected the participants' narratives.

Three master themes emerged from this analysis: Feelings about food waste, reasons for food waste, and how to avoid or eliminate food waste .

The various themes emerged are listed in Table 1.

**Table 1:** Overview of the master themes and sub-themes

Sr. No.	Question	Master Themes	Sub-Themes
1	Feelings about food waste	Emotional distress	Guilt
			Emotional distress
			Sin
2	Reasons for Food Waste	Family culture	Family values
			Miscalculations
			Lack of appreciation
			Food preferences
		Rising working class	Working class
			Children and working adults
			Time constraints
			Storage facility
			Bulk purchase and stock
		Unthemed	Special occasions
3	How to avoid or eliminate food waste	Improving family values	Aligning value systems
			Consumption habits
			Enhancing awareness and appreciation
			Purchasing or cooking
			Initiatives for children
		Finding alternative ways of consumption of food	Re-use
			Food sharing
			Managing storage
		Unthemed	Managing special occasions

**Theme1: Emotional Distress**

This theme revolved around the emotional distress associated with food wastage. All the participants who took part in the study expressed significant concern regarding food waste in Indian households. It can be concluded that nobody intends to waste food, but in many instances, like problems in accurate estimation, deep storage, power failure and food preferences and consumption habit, it is unavoidable. The finding was in line with the Attiq et al. (2021) where ‘anticipated guilt’ and ‘awareness of consequences’ was found to be significant driver of reuse and reduce food waste in the context of United States. Russell et al. (2017) found that ‘negative emotions’ lead to greater intentions to reduce food waste.

**Theme2 (a and b): Family Culture and Rising Working Class**

This theme is based on an inquiry into the reasons for food waste. The analysis revealed two primary themes: family culture and the growth of the working class, with special occasions being an exception. At the core of food waste in Indian households lies the issue of family culture. Another theme that emerged in the research is the rising working class, which can be categorized under sub-themes of the working class, children and working adults, time constraints, storage facility, and bulk purchases. This finding is closely related with Jamaludin et al. (2022) study in which ‘food management practices’ were found to significantly affect food waste in Malaysia.

**Theme3 (a and b): Improving Family Values and Finding Alternative Methods of Consumption**

The researchers identified two primary themes during their investigation of methods for reducing or eliminating food waste: improving family values and finding alternative methods of consumption. The theme of improving family values involved enhancing individuals' values systems, consumption habits, awareness, and appreciation of food, purchasing and cooking in appropriate quantities, as well as implementing initiatives for children. On the other hand, the theme of finding alternative methods of consumption focused on effectively managing storage, sharing food, and reusing food. Several participants emphasized the importance of reusing leftover food as a method of reducing food waste. The finding is in line with Filimonau et al. (2022), observation that religious and family values had a significant impact on intention to waste food.

## Discussion

This study aimed to examine the experience of food waste in Indian households and understand the extent of food waste in these households. Through an idiographic investigation, the study revealed that food waste is a prevalent issue in Indian households. During the interviews, respondents provided their daily/weekly food waste estimates. From their estimates, authors concluded that it is almost close to the study of UNEP (2021).

The study also examined the emotions and attitudes of Indian households toward food waste. The findings revealed that food waste elicits feelings of guilt and sadness for most respondents. Participants expressed that wasting food feels like committing a sin, and some even described it as emotionally painful. Despite its prevalence in Indian households, food waste appears to be a source of discomfort, and they are actively seeking ways to reduce or eliminate it. The study identified various reasons for food waste in Indian households. These include a decline in family values, miscalculations, the rise of the working class, lack of appreciation for food, special occasions, storage facilities, bulk purchases, food preferences, and time constraints. Among households where both partners work, lack of time and appreciation for food preferences emerged as the leading causes of food waste.

The study suggests several solutions to reduce, minimize, or eliminate food waste, such as improving value systems and consumption habits, enhancing awareness and appreciation, managing special occasions better, sharing food, and managing food storage. Participants recommended teaching children to appreciate food and avoid waste from an early age. They also suggested avoiding overcooking, miscalculations, and over-ordering during special occasions, and reverting to traditional Indian culture and practices such as appreciating the efforts of farmers, eating with family members, and using food waste as feed for livestock.

The study's focus on kitchen-related food waste in Indian households may limit its scope. Respondents also noted wasting food during special occasions or dining out, which were not included in this research. Future studies could explore these areas to gain a more comprehensive understanding of the issue.

## Conclusion

In summary, the study sheds light on the prevalence of food waste in Indian households, the related attitudes and emotions, and the reasons behind it. It also offers practical solutions for reducing, minimizing, or eliminating food waste, which could serve as guidelines for individuals, households, and policymakers. However, further research is needed to validate the findings by examining how these may vary in various geographical regions of India or households of different social classes. Mixed methods or quantitative research could produce comparable results and provide more comprehensive insights.

## Data Availability

All data generated or analysed during this study are included in this published article.
