# Correction to: Food waste in Indian households: status and potential solutions

**DOI:** 10.1007/s11356-023-31210-3

**Published:** 2023-11-27

**Authors:** Samant Shant Priya, Sushil Kumar Dixit, Sajal Kabiraj, Meenu Shant Priya

**Affiliations:** 1 Lal Bahadur Shastri Institute of Management (LBSIM), New Delhi, Delhi India; 2 Lal Bahadur Shastri Institute of Management (LBSIM), New Delhi, India; 3 School of Business, Design and Technology, Häme University of Applied Sciences Ltd. (HAMK), Valkeakoski, Finland; 4 School of Business, Galgotias University, Greater Noida, India

**Correction to: Environmental Science and Pollution Research**

10.1007/s11356-023-31034-1

Meenu Shant Priya is only affiliated to affiliation 4.

The Original article has been corrected.

